# An Interesting and Horribly Wondrous Sight

**DOI:** 10.3201/eid2704.AC2704

**Published:** 2021-04

**Authors:** Byron Breedlove

**Affiliations:** Centers for Disease Control and Prevention, Atlanta, Georgia, USA

**Keywords:** art science connection, emerging infectious diseases, art and medicine, about the cover, an interesting and horribly wondrous sight, eruption of the volcano Vesuvius, Johan Christian Dahl, volcanoes, public health, surveillance, high-consequence pathogens, viruses, Middle East respiratory syndrome coronavirus, Nipah virus, Ebola, Marburg hemorrhagic fever, Crimean-Congo hemorrhagic fever, Rift Valley fever, anthrax, leprosy, melioidosis, rabies, smallpox

**Figure:**
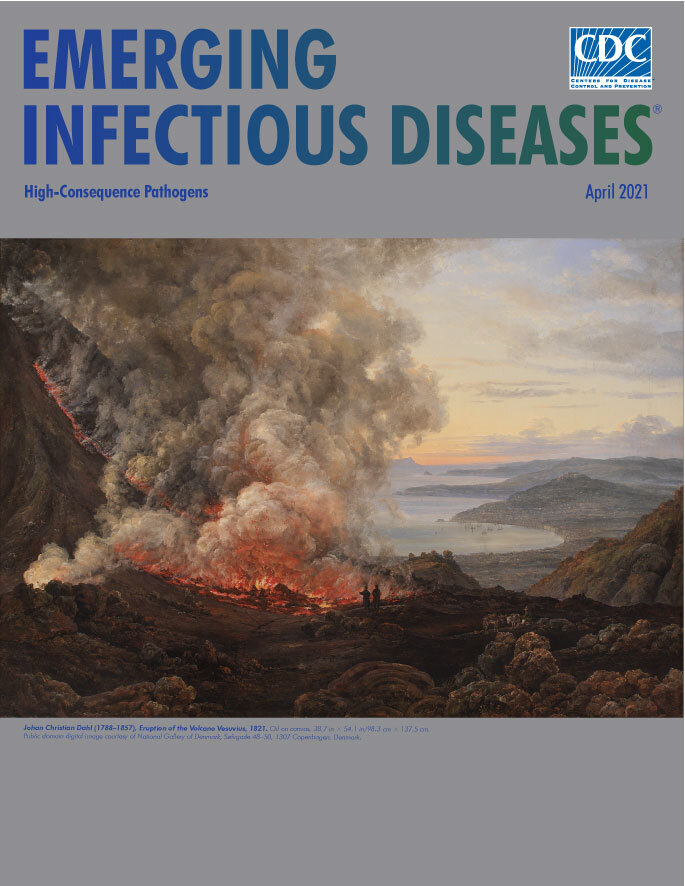
**Johan Christian Dahl (1788–1857), *Eruption of the Volcano Vesuvius*, 1821.** Oil on canvas, 38.7 in × 54.1 in/98.3 cm × 137.5 cm. Public domain digital image courtesy of National Gallery of Denmark, Sølvgade 48–50, 1307 Copenhagen, Denmark.

Volcanoes―active, dormant, and extinct―are found on every continent on Earth. Many lay submerged below the oceans, and others exist as islands. Volcanic explosions, upheavals, and lava flows, coupled with eons of weathering, have formed mountains and plateaus, created craters, and etched valleys. Approximately 80 percent of Earth’s surface was created by volcanic activity. The United States Geological Survey notes, “Gaseous emissions from volcanic vents over hundreds of millions of years formed the Earth’s earliest oceans and atmosphere, which supplied the ingredients vital to evolve and sustain life.” 

Ancient to modern eyewitness accounts document the devastation and spectacle associated with volcanic eruptions. For centuries, artists have depicted in their works both the sublime beauty and unthinkable destruction of volcanic eruptions. Among them is the Norwegian painter Johan Christian Claussen Dahl. Considered the first great Romantic painter in Norway and among the greatest European artists of all time, Dahl traveled to Italy during the fall of 1820. He visited Naples in late December of that year and observed firsthand Vesuvius erupting, an event he called an “interesting and horribly wondrous sight.” This month’s cover image, *Eruption of the Volcano Vesuvius, 1821,* is the first in a series of paintings he created after that experience. 

Dahl’s up-close portrayal reverses the more common artistic perspective of placing volcanoes in the background. In this painting, clouds of smoke and steam flecked with lava billow and whirl skyward from the glowing red fissures and caldera of Mount Vesuvius, obscuring much of the sky and water in the left half of the painting. Near the edge of the crater, a pair of visitors (perhaps one represents Dahl) appear as tiny silhouettes and convey a sense of scale. A panoramic view of Naples receding in the background and hugging the shore of its namesake gulf in the Mediterranean Sea fills the right side of the painting. More mountain ridges rise and jut into the bay under a panoramic, calm sky. Nearly hidden in the foreground, a local guide waits and watches, clutching the tethers of a pair of donkeys.

Art historian Maia Heguiaphal writes, “The vast smoke coming out of the incandescent lava immediately attracts the viewer’s eyes, but the background of the volcano intensifies the effect of destruction carried by the eruption. Dahl makes us see the landscape that the volcano will destroy: The lava flows in its direction. We are therefore confronted with the imminent destruction of idyllic nature.” 

Worldwide, approximately 1,500 volcanoes are potentially active, and on any given day, about a dozen may be erupting. The World Health Organization estimates that during 1998−2017, volcanic activities and the wildfires they spawned affected 6.2 million people and caused nearly 2,400 deaths. Geosciences professor Erik Klemetti notes, “With our modern ability to monitor volcanoes in many remote locations thanks to satellites, and the speed with which news travels around the globe today, an eruption that might have gone unnoticed 100 years ago is bound to make headlines in 2018. The world is not more volcanically active, we’re just more volcanically aware.”

Volcanic eruptions are among the most dramatic, unpredictable, and dangerous threats from Mother Nature. But unseen threats, arriving more stealthily via emerging and reemerging high-consequence pathogens, are much more deadly. High-consequence pathogens cause diseases that typically have a high case-fatality rate, are often difficult to recognize and detect rapidly, may spread rapidly and cause epidemics, and lack effective measures for prophylaxis or treatment. For example, the 2014−2016 Ebola outbreak in West Africa alone caused more than 11,000 deaths. 

Among the diseases caused by high-consequence pathogens are Middle East respiratory syndrome, Nipah virus infection, monkeypox, and a cluster of hemorrhagic fever diseases (in addition to Ebola, including Marburg hemorrhagic fever, Crimean-Congo hemorrhagic fever, and Rift Valley fever). Such pathogens are also responsible for many diseases that have been known for centuries, such as anthrax, melioidosis, rabies, and smallpox. 

Although many pathogenic agents may be circulating at any given time, disease outbreaks (or even small numbers of cases) caused by high-consequence pathogens can have serious public health, economic, and even security consequences. That point is underscored by the current COVID-19 pandemic. Although the case-fatality rate associated with SARS-CoV-2 infection is lower than that of many other viruses (i.e., is less deadly at the individual level), the enormous number of persons infected and the ability of SARS-CoV-2 to spread rapidly results in many more deaths.

CDC-based scientists Belay and Monroe state that “ongoing surveillance and public health research of high-consequence pathogens are critical for identifying their natural reservoirs, developing diagnostic tests, and devising appropriate control and prevention measures.” Though the world is now more pathogenically aware than ever before, high-consequence pathogens are, like volcanoes, predictably unpredictable, and they are an ongoing public health concern. 
